# Supplementary material for: Metabolic Profiling, Chemical Composition, Antioxidant Capacity, and In Vivo Hepato- and Nephroprotective Effects of Sonchus cornutus in Mice Exposed to Cisplatin
Source: Antioxidants (Basel). 2022 Apr 22;11(5):819. doi: 10.3390/antiox11050819 (PMC9137627; doi:10.3390/antiox11050819)
Supplement: Supplementary file 1 [file antioxidants-11-00819-s001.zip › antioxidants-1683097-supplementary.pdf]

Metabolic Profiling, Chemical Composition, Antioxidant Capacity and In vivo Hepato- and Nephroprotective Effects of *Sonchus cornutus* in Mice Exposed to Cisplatin

Supplementary materials for detailed methodology

1. **Figure S1.** Chromatogram of LC- MS/MS analysis of crude extract of *Sonchus cornutus* (positive mode)
2. **Figure S2.** Chromatogram of LC- MS/MS analysis of crude extract of *Sonchus cornutus* (negative mode)
3. **Table S1.** GenBank accession numbers, primer sequences and annealing temperatures of the assessed genes.

LC-MS/MS Metabolic Profiling

High performance liquid chromatography- triple time-of-flight tandem mass spectrometry (HPLC/Triple-TOF-MS/MS) was established as mentioned before [1,2]. The ethanolic crude extract of *S. cornutus* was dissolved in a mixture of water: methanol: acetonitrile (50:25:25) to afford a solution at a concentration of (0.5 mg/mL). The prepared solution was centrifuged, then 50 μL were picked up and completed to 1000 μL with water: methanol: acetonitrile (50:25:25). Ten μL was injected in both the positive and negative modes. The LC/Triple-TOF-MS/MS analysis was conducted using an ExionLC system (AB Sciex, Framingham, MA, USA) with an autosampler system, an in-line filter disks pre-column (0.5 μm × 3.0 mm, Phenomenex, Torrance, CA, USA), and an X select HSS T3 column (2.5 μm, 2.1 × 150 mm, Waters Corporation, Milford, MA, USA) sustained at 40 °C. The mobile phase consisted of 5 mM ammonium format buffer in 1% methanol with the pH adjusted to 3.0 and 8.0 for positive and negative modes. The mobile phase was gradually eluted by increasing the concentration of the acetonitrile within 20 min, followed by a constant period of 4 min, and finally, a decrease in the acetonitrile concentration within 3 minutes with a constant flow rate of 0.3 mL/min. This compartment was connected to a Triple TOF™ 5600+ system (AB SCIEX, Concord, Canada) to detect the MS/MS transitions of the analytes. The detected metabolites were recognized by means of their *m/z* and MS/MS transitions compared to those found in recorded databases. The mass accuracy was calculated as follows: [measured mass-expected mass/expected mass] × 10^6 and expressed in parts per million (ppm) error [3-5]. Moreover, the MZmine ID, retention time, adduct formula, and molecular formula were detected.

1. Abdel-Hamed, A.R.; Mehanna, E.T.; Hazem, R.M.; Badr, J.M.; Abo-Elmatty, D.M.; Abdel-Kader, M.S.; Goda, M.S. *Plicosepalus acacia* extract and its major constituents, methyl gallate and quercetin, potentiate therapeutic angiogenesis in diabetic hind limb ischemia: HPTLC Quantification and LC-MS/MS metabolic profiling. *Antioxidants* **2021**, *10*, 1701. DOI: 10.3390/antiox10111701

2. Goda, M.S.; Nafie, M.S.; Awad, B.M.; Abdel-Kader, M.S.; Ibrahim, A.K.; Badr, J.M.; Eltamany, E.E. In vitro and in vivo studies of anti-lung cancer activity of *Artemesia judaica* L. crude extract combined with LC-MS/MS metabolic profiling, docking simulation and HPLC-DAD quantification. *Antioxidants* **2022**, *11*, 17. DOI: 10.3390/antiox11010017

3. Abdelhameed, R.F.A.; Habib, E.S.; Goda, M.S.; Fahim, J.R.; Hassanean, H.A.; Eltamany, E.E.; Ibrahim, A.K.; AboulMagd, A.M.; Fayez, S.; El-kader, A.M.A.; Al-Warhi, T.; Bringmann, G.; Ahmed, S.A.; Abdelmohsen, U.R. Thalassosterol, a new cytotoxic aromatase inhibitor ergosterol derivative from the Red Sea seagrass *Thalassodendron ciliatum*. *Mar. Drugs* **2020**, *18*, 354. DOI: 10.3390/md18070354

4. Eltamany, E.E.; Elhady, S.S.; Goda, M.S.; Aly, O.M.; Habib, E.S.; Ibrahim, A.K.; Hassanean, H.A.; Abdelmohsen, U.R.; Safo, M.K.; Ahmed, S.A. Chemical composition of the Red Sea green algae *Ulva lactuca*: Isolation and in silico studies of new anti-COVID-19 ceramides. *Metabolites* **2021**, *11,* 816. DOI: 10.3390/metabo11120816

5. Elhady, S.S.; Habib, E.S.; Abdelhameed, R.F.A.; Goda, M.S.; Hazem, R.M.; Mehanna, E.T.; Helal, M.A.; Hosny, K.M.; Diri, R.M.; Hassanean, H.A.; Ibrahim, A.K.; Eltamany, E.E.; Abdelmohsen, U.R.; Ahmed, S.A. Anticancer effects of new ceramides isolated from the Red Sea red algae *Hypnea musciformis* in a Model of Ehrlich ascites carcinoma: LC-HRMS analysis profile and molecular modeling. *Mar. Drugs* **2022**, *20*, 63. DOI: 10.3390/md20010063

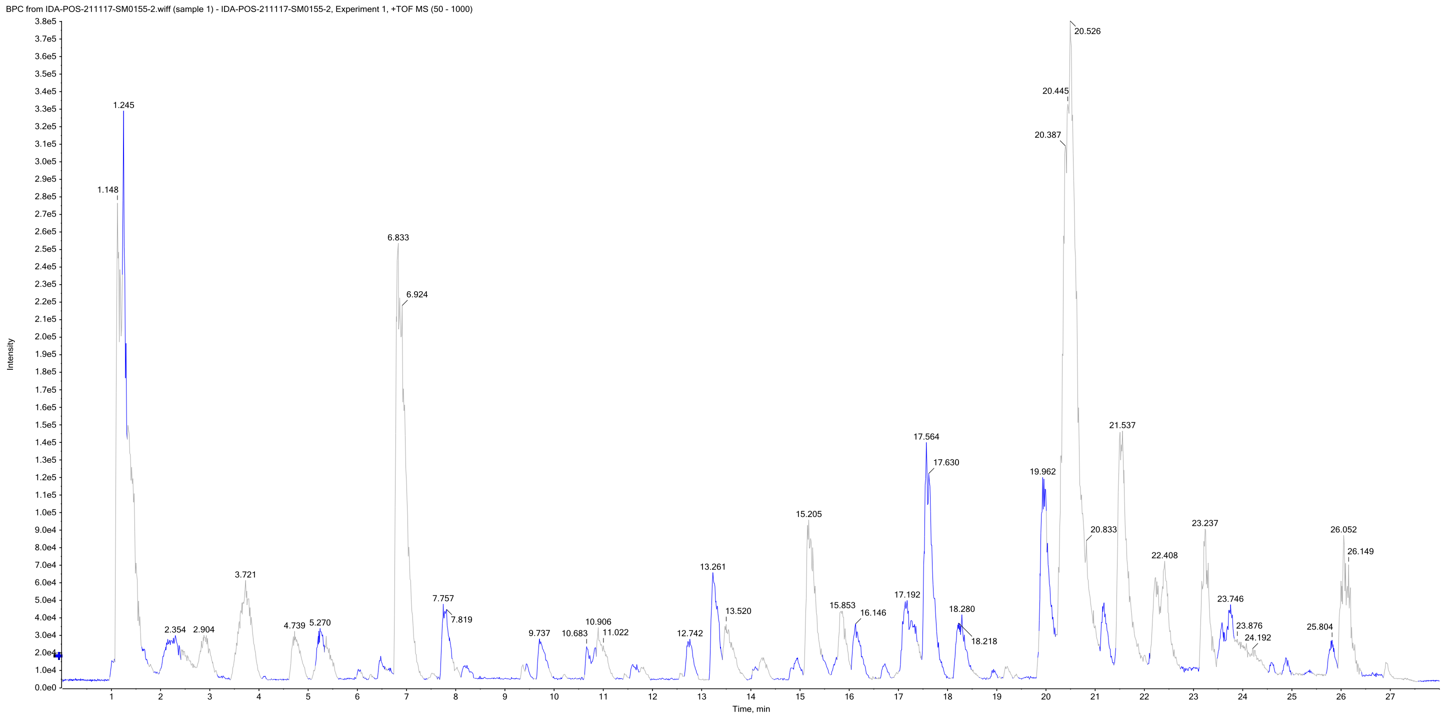

Figure S1. Chromatogram of LC-MS/MS analysis of crude extract of *Sonchus cornutus* (positive mode).

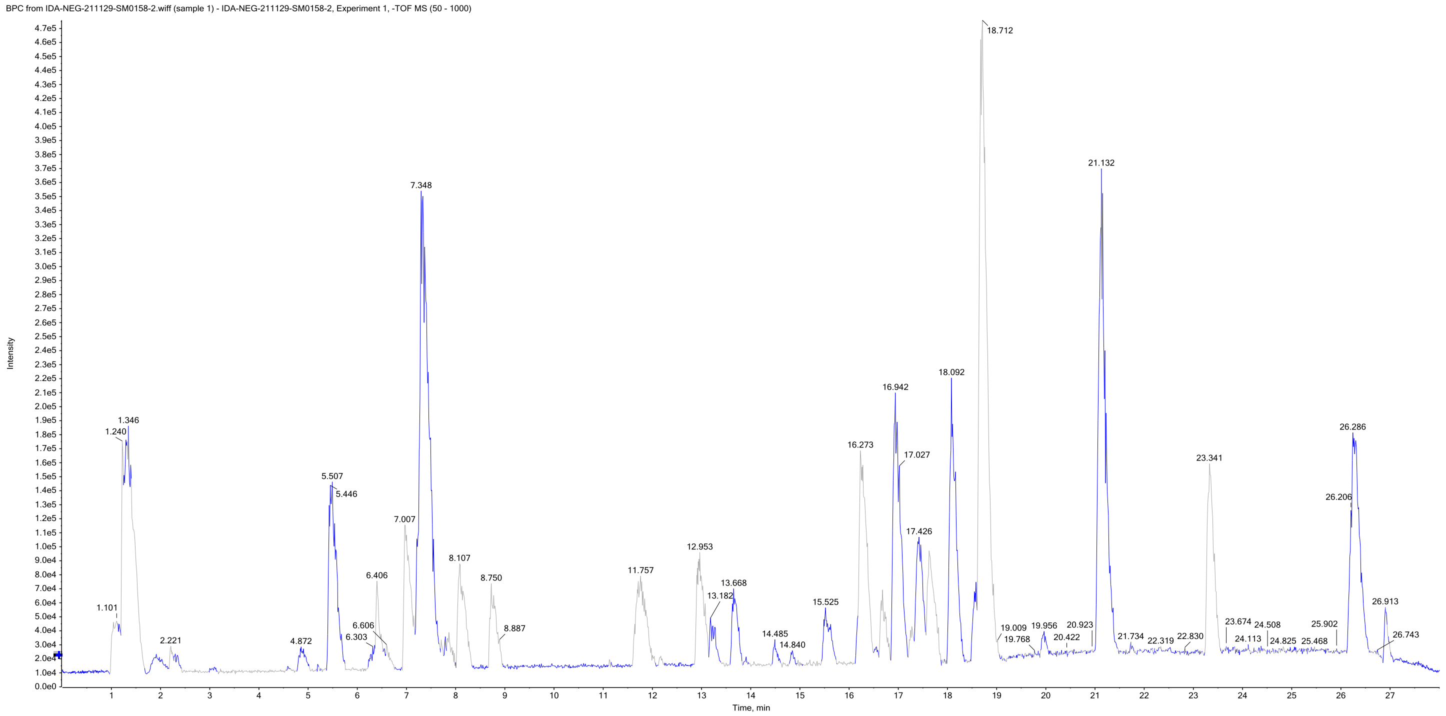

Figure S2. Chromatogram of LC-MS/MS analysis of crude extract of *Sonchus cornutus* (negative mode).

Table S1. GenBank accession numbers, primer sequences and annealing temperatures of the assessed genes.

GenBank Accession No.	Gene	Primers	Annealing temperature
NM_009045.5	NF-κB	Forward: 5'-CAATGGCTACACAGGACCA-3'<br>Reverse: 5′-CACTGTCACCTGGAACCAGA-3′	52ºC
NM_013693.3	TNF-α	Forward: 5′-TCTACTGAACTTCGGGGTGATCG-3′<br>Reverse: 5′-TGATCTGAGTGTGAGGGTCTGGG-3′	56ºC
NM_010902.4	Nrf2	Forward: 5′-CTCTCTGGAGACGGCCATGACT-3′<br>Reverse: 5′-CTGGGCTGGGGACAGTGGTAGT-3′	58ºC
NM_007393.5	β-actin	Forward: 5′-ACGGCCAGGTCATCACTATTG-3′<br>Reverse: 5′-CAAGAAGGAAGGCTGGAAAAGA-3′	52ºC
